# Understanding the Influence of Environment on Adults’ Walking Experiences: A Meta-Synthesis Study

**DOI:** 10.3390/ijerph13070731

**Published:** 2016-07-20

**Authors:** Sara Dadpour, Jahanshah Pakzad, Hamidreza Khankeh

**Affiliations:** 1 Department of Urban Planning & Design, Shahid Beheshti University, Daneshjoo Blvd, Evin, Tehran 19839-63113, Iran; s_dadpour@sbu.ac.ir (S.D.); J-Pakzad@sbu.ac.ir (J.P.); 2 Research Center in Emergency and Disaster Health, University of Social Welfare and Rehabilitation Sciences (USWR), Koudakyar St., Daneshjoo Blvd, Evin, Tehran 19857-13834, Iran; 3 Department of Clinical Science and Education, Karolinska Institute, Södersjukhuset (KI SÖS) Sjukhusbacken 10, Stockholm 118 83, Sweden

**Keywords:** walking, environment, systematic review, meta-synthesis, adults

## Abstract

The environment has an important impact on physical activity, especially walking. The relationship between the environment and walking is not the same as for other types of physical activity. This study seeks to comprehensively identify the environmental factors influencing walking and to show how those environmental factors impact on walking using the experiences of adults between the ages of 18 and 65. The current study is a meta-synthesis based on a systematic review. Seven databases of related disciplines were searched, including health, transportation, physical activity, architecture, and interdisciplinary databases. In addition to the databases, two journals were searched. Of the 11,777 papers identified, 10 met the eligibility criteria and quality for selection. Qualitative content analysis was used for analysis of the results. The four themes identified as influencing walking were “safety and security”, “environmental aesthetics”, “social relations”, and “convenience and efficiency”. “Convenience and efficiency” and “environmental aesthetics” could enhance the impact of “social relations” on walking in some aspects. In addition, “environmental aesthetics” and “social relations” could hinder the influence of “convenience and efficiency” on walking in some aspects. Given the results of the study, strategies are proposed to enhance the walking experience.

## 1. Introduction

A considerable number of adults throughout the world suffer from insufficient physical activity. In 2010, about 23% of adults and in 2012, about 31% of adults over the age of 15 years were physically inactive [1,2]. Insufficient physical activity in adults (less than 150 min of intermediate walking per week) increases the risk of death and non-communicable diseases such as cardiovascular disease, stroke, diabetes, and some types of cancer [2].

Walking is the most usual type of physical activity for most people [3,4] and can be continued as a person ages to reduce the physiological risks of low physical activity [5]. Providing an encouraging environment for walking could encourage many people to increase their physical activity, even those of low economic status [6,7]. Apart from the physical benefits, walking has psychological benefits as well [8]. For example, it reduces symptoms of depression [9,10], reinforces thinking and creativity [11], and provides a means for the expression of positive feelings [12]. In addition, as a mode of active transportation in the daily lives of individuals, walking can reduce dependence on hydrocarbon sources of energy and pollution of the environment [13]. Consequently, walking should be studied specifically because of its broad influence on public health.

Understanding the factors which impact on the walking experience is necessary for providing conditions and an environment that encourages walking. Identifying factors influencing the walking experience is a multidisciplinary subject and is used in the fields of urban design and planning, health and transportation [3,4,14]. Studies on the subject have changed over the past few years. Previously, the emphasis was on individual factors influencing walking [14,15]; however, for about two decades, various scientific disciplines have focused on the influence of the environment (especially the built environment) on walking [4]. For example, the influence of distance to destinations on walking has been emphasized in various studies, such as Cauwenberg et al. [16], Cerin et al. [17] and King et al. [18]. It is necessary, then, to review and compare the results of studies conducted in different contexts to reach a more comprehensive perspective about the influence of the environment on walking.

Studies conducted on the influence of environmental factors on walking are mainly quantitative, while qualitative studies are complementary to quantitative studies [19]. Qualitative studies provide a deeper understanding of social phenomena [19], such as walking as experienced subjectively in relation to the physical and social context. Qualitative studies identify the different meanings of participant interactions with the environment [19,20,21]; therefore, they can improve knowledge about how environmental factors influence walking [21].

Studies have recommended that specific types of physical activity, such as walking, should be studied separately in relation to the context [4,22]. It has also been shown that the influence of the environment on walking and cycling is more important than for other types of physical activity [23]. Some studies have identified walking as a unique type of physical activity [24,25,26]. Some other studies have referred to different environmental factors influencing walking compared to physical activities like cycling [7,23,27]; however, studies have primarily investigated walking along with other physical activities or other modes of transportation. It is necessary to study walking separately from other types of physical activity. There are some systematic reviews of qualitative studies emphasizing the influence of the environment on physical activity in general rather than on walking in particular (such as Moran et al. [21], Siddiqi et al. [28], and Allender et al. [29]). The systematic queries made by the authors have found no comprehensive study of qualitative research conducted on the influence of the environment that focuses only on walking. 

The present study is a systematic review of qualitative studies that explores the influence of the environment on walking separately from other physical activities or modes of transportation. The purposes of this systematic review is to identify the environmental factors influencing walking and understand how they impact on walking based on the experiences of adults. Since the environmental requirements of adults differ from those of children and adolescents [30], only adults between the ages of 18 and 65 were examined.

## 2. Methods

The present study is a meta-synthesis of qualitative studies on the influence of the environment on walking through a systematic review. Meta-synthesis of qualitative studies is a parallel methodology to meta-analysis of quantitative studies. In meta-synthesis, the findings of former studies on a subject are interpreted and synthesized to reach a more comprehensive and deeper understanding of the subject [31,32]. The systematic review was conducted on the basis of Preferred Reporting Items for Systematic Reviews and Meta-Analyses (PRISMA) statement [33,34].

During the first phase, a systematic search of different databases was carried out to find qualitative and quantitative systematic reviews about the influence of the environment on walking. The purpose of this phase was to ensure that the study has not been duplicated and to select proper keywords for the main search. During the second phase, a comprehensive and systematic search was conducted of related databases from different disciplines. Given the eligibility criteria, the papers identified were checked by reading the titles, abstracts, and full texts. Those failing the criteria were eliminated from the study. The studies selected met all inclusion criteria and did not meet any of the exclusion criteria. The eligibility criteria were established before searching and were discussed until agreement was reached. During the third phase, the eligible papers were assessed for risk of bias on the basis of a qualitative framework for assessing qualitative studies and those failing the assessment were eliminated from the study. Only studies qualified for meta-synthesis were selected. During the fourth phase, the general characteristics of the selected studies and their findings sections were extracted. During the fifth phase, the findings of the studies were interpreted and synthesized through qualitative content analysis.

### 2.1. Eligibility Criteria

#### 2.1.1. Inclusion Criteria

- Studies having a qualitative research design.
- Studies in which the influence of environment on walking was mentioned in the findings section.
- Studies focusing on walking or analyzing walking separately from other modes of transportation or types of physical activity.
- Studies in which the participants were 18–65 years of age or the average age was within these limits.

#### 2.1.2. Exclusion Criteria

- Studies which only investigated the influence of non-physical environmental factors on walking. The reason for selection of this criterion was that many quantitative studies have shown the influence of the built environment factors on walking.
- Studies that investigated the influence of environment on walking under conditions of intervention experience. The reason for this was the requirement that the influence of environmental factors on walking could be studied in natural conditions. Specific environmental factors should not be emphasized.
- Studies focused on patients or people with health conditions influencing walking, such as rehabilitation after stroke.
- Studies focused on non-public spaces such as offices.

### 2.2. Information Resources

**Figure 1:**
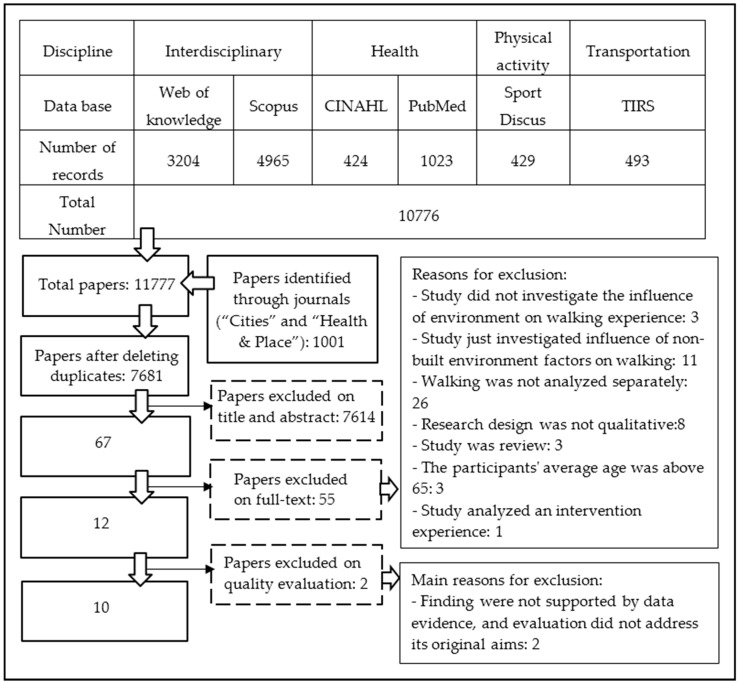
Diagram for access to relevant papers.

Seven databases of different disciplines related to the influence of the environment on walking and two journals, i.e., “Cities” and “Health and Place” were queried. The impact factors of both journals were above 1.5. The databases were Web of Knowledge, Scopus, PubMed, CINAHL, TIRS, Sport Discus, and RIBA (Figure 1). The RIBA database was queried separately because of its search and software limitations; however, no paper suitable for synthesis was found in this database. Both journals were accessed through ScienceDirect.

### 2.3. Search

The search keywords were categorized into three groups (Table 1) and were chosen to attain the highest number of potentially eligible studies. To choose the keywords, qualitative and quantitative systematic review studies were systematically searched and their keywords were used. The keywords of the qualitative studies were obtained from a study by Shaw et al. [35].

Similar keywords relating to a group of words (a table row in Table 1) were linked with “or” and the keyword groups were linked with “and”; these included walking, physical environment, and qualitative keywords.

The searches were limited to academic and peer-reviewed papers with abstracts written in English. The time period for searching was limited to 1990 onward because the influence of environmental factors on walking and physical activity has received increased attention since that time [4,15].

### 2.4. Selection of Studies

All searches were conducted from 9 November to 17 November 2015 by the first author. A total of 11,777 papers were obtained from the databases and journals. After eliminating duplicate papers and checking paper titles, abstracts, and full-text, 12 papers were identified as meeting the specified criteria (Figure 1).

The risk of bias was evaluated in 12 papers using the qualitative framework proposed by Spenser et al. [36]. Ten of these were identified as meeting the required quality. To find relevant new papers, the method of backward tracing was used for high-quality studies, but no new papers were found. Two of the final papers [37,38] were about a single study.

### 2.5. Data Extraction

The findings sections of each study was extracted for synthesis. Other parts were extracted for comparison and are simplified and summarized in Table 2. All studies were conducted in urban and/or suburban areas in Australia [39,40,41], Europe [8,30,37,38,42,43], or North America [44]. The scales of the study fields varied from the neighborhood [40,43] to beyond city-wide [30,39,41,42]. The studies by Bostock [40] and Burgoyne et al. [43] were conducted in low income areas. The participants of one study [40] were women from social minorities and the participants of other studies were both men and women.

### 2.6. Data Analysis

Qualitative content analysis was used to analyze the results of the studies. The results of each study were first studied and then recoded. The explicit and implicit concepts presented were the basis for the interpretation and recoding of the new concepts. At this point, an attempt was made to consider and obtain the maximum number of new concepts [45]. In the next step, the concepts derived from the studies were categorized by constant comparison analysis. Themes and subthemes were developed based on the similarities and differences of the concepts [46,47,48].

## 3. Findings

The main themes of “safety and security”, “environmental aesthetics”, “social relations”, and “convenience and efficiency” were derived from the analysis (Table 3). Each theme and its sub-themes and concepts are described in detail below. The meanings of the sub-themes are also explained.

### 3.1. Safety and Security

#### 3.1.1. Sense of Insecurity

Fear of crime constituted the meaning of sense of insecurity. The absence of others or poor lighting at nighttime, closed shops, vacant land, and parking lots influenced the sense of insecurity [30,41]. It was believed that white light was more effective than yellow light in illuminating walking spaces [30]. Vagrants, juvenile delinquents, and antisocial behavior, such as alcohol and drug abuse, were other contributors to the feeling of insecurity [30,43]. The presence of traffic in proximity to gang hangouts reduced the sense of insecurity [43]. Walking with a dog also reduced feelings of insecurity [41,44]. Some participants, particularly women, mentioned the feeling of insecurity as one of the most important barriers to walking [30].

#### 3.1.2. Sense of Inadequate Safety

Feeling a lack of safety was identified as a fear of accidents with cars or bicycles and being attacked by animals. Crossing wide streets or roundabouts, crossing streets where there was a possibility of cars turning around, and the absence of pedestrian traffic lights with counters resulted in the feeling of inadequate safety [30]. Improper conduct by drivers and the high speed of cars and bicycles were reasons of the sense of inadequate safety [30,39,40,41,43], which were emphasized in walking with children in low income areas [40,43]. Streets that were either too wide or too narrow and car and bicycle lanes that were not separate from sidewalks were factors contributing to the sense of inadequate safety [30].

The presence of horses [42,43] and wandering dogs [30,41,43] were the other reasons for participants’ sense of low safety. Some participants believed walking with dogs reduces feelings of low safety [43]; while wandering dogs were a cause of feelings of low safety in dog walking [41].

### 3.2. Environmental Aesthetics

#### 3.2.1. Built Environment Aesthetics

Features of the built environment which were sensually and perceptually pleasing to participants or which were displeasing and attracted negative attention constituted the meaning of built environment aesthetics. Built environment aesthetics were important for both transportation and recreational walking. Picturesque scenery, like an architecturally beautiful building or wall art, encouraged participants to walk [30,42]. Seeing shops in deteriorating condition discouraged walking [37]. Environmental variety of colors and forms encouraged walking [30,39,41,42]. Variety in the design of walking spaces was believed to help attract a larger and more varied group of people to walking [42]. Experiencing a detailed environment, such as the presence of different types of urban furniture was an incentive to walk [30,39,42] and an environment without these details, such as long bare walls, made walking unpleasant [30].

Order, integrity and legibility in the environment encouraged walking [30,42], although excessive order and an excess of route signs and symbols for pedestrians were not pleasant for recreational walking [42].

Uncleanliness such as unpleasant odors, inefficient trash collection, poor drainage systems, the presence of dog feces, broken windows, and abandoned tools discouraged walking [30,38,40,41,42,43]. Uncleanliness focused participant attention on the environment at the expense of contemplation and concentration during the walking experience [38].

#### 3.2.2. Natural Elements

Features of the natural environment which pleased participants sensually and perceptually formed the meaning of natural elements. Green elements, like trees and plants, were pleasant and encouraged walking while the absence of trees discouraged walking [30,41]. The presence of water in the form of lakes, rivers and fountains encouraged walking [41,42]; however, natural water elements, like ponds in parks, were found to be problematic for dog walking because they presented control problems for the dog owners [41].

Natural light and fresh air were also among the elements encouraging walking [8,30]. Visiting scenic landscapes, like hills, encouraged walking [8,42,43] and increased relaxation and contemplation [8,42].

### 3.3. Social Relations

#### 3.3.1. Being with Others

Being with others was explored as the way participants experienced themselves in relation to the persons around them while walking. Walking in crowded places was unpleasant [30,42]. In recreational walking, the presence of a large number of people decreased the sense of quiet and relaxation [42]. Uncrowded places allowed contemplation and had psychological benefits [38].

The company of others while walking was an encouraging factor [39,41,42,43]; however, the company of children influenced the walking experience differently. Viewing beautiful natural scenery helped the participants enjoy walking with others [8]. Dog owners referred to their dogs as companions that kept them from feeling lonely while walking [41]. Walking the dog encouraged all family members to walk together [44].

Studies have reported that participants experienced seeing others (strangers or acquaintances) and talking with them as a pleasant activity while walking [8,30,37,39,41,42]. Wide sidewalks, a sense of being in a local area, security and safety, environmental aesthetics, and separation of the walking space from heavy traffic were incentives for social interactions in a walking environment [39]. Meeting other people walking their dogs was an incentive for dog walking [41]. Facilities for interacting with others like benches and barbecues encouraged walking [30,41]; yet meeting people was not always a pleasant experience. An unfriendly ambience on the street contributed to an unpleasant walking experience. Walking among people who did not walk in a straightforward direction because they were constantly checking their mobile phones was also an annoying experience [37].

#### 3.3.2. Public Perception

Common viewpoints and perceptions among community members which influenced their walking experiences constituted the meaning of public perception. Among parents, the social pressure of other parents to walk more with their children was a factor influencing walking with children. These parents felt guilty about driving their children to their destinations [39]. The prevalence of the car culture—the public preference for using cars instead of other transportation modes—was perceived as a barrier to walking [42].

A sense of abandonment was also a factor discouraging walking in poor areas. The reason for this was the lack of neighborhood walking facilities or their inadequate maintenance [40,43]. The lack of support by local authorities, including the absence of financing and the absence of supportive laws and regulations were reasons for the lack of proper walking facilities. This led to a sense of abandonment by local authorities [43].

### 3.4. Convenience and Efficiency

Environmental factors which impacted on the convenience and comfort of participants while walking formed the meaning of convenience and efficiency. This also referred to environmental factors which influenced on how efficient and logical participants found walking to be in comparison with alternative activities, such as cycling.

#### 3.4.1. Time of Walking

Short walkable distances to a destination encouraged transportation walking [30,37,39,41]. When several choices to reach a destination were available, the shorter distance was preferred for transportation walking [30,37]. Long distances to services, such as medical, shopping, or occupational centers or public transportation stations discouraged walking to such destinations [8,40]. The difficulty of walking long distances with small children to shop was emphasized [40]. Walking a long way down a very long street caused the destination to be perceived as farther away and made transportation walking unpleasant [30]. Spending less time on walking was not desirable in all conditions. Enjoying natural elements along the way encouraged spending more time and selecting longer routes for walking [30,37]. Relaxation and the joy of observing nature decreased the speed of walking and increased the time spent walking [8]. An unfriendly social ambience and lack of built environmental aesthetics on a particular street resulted in choosing another street, although it increased the time walked. An unfriendly social ambience was experienced as a barrier to enjoyable slow walking [37]. Spending more time for recreational walking was not problematic in comparison with transport walking [8].

The necessity of making lengthy and numerous stops while walking discouraged walking for transportation. A long wait for traffic lights to turn green was one factor causing stops during walks. Lack of harmony between adjacent traffic lights for pedestrians and multiple junctions were additional factors which required lengthy and numerous stops [30,38].

The maximum time limit deemed feasible for walking as transportation had a negative impact on walking. Participants accustomed to using cars had lower maximum time limits for proper and efficient walking [8,30]. The sense of a shortage of time was attributed either to lifestyle or mismanagement of time [8,42]. Shortage of time was a reason for preference for other modes of transportation, like cycling. Moreover, walking with a shortage of time was experienced as being unpleasant [8]. The time condition of walking influenced on its pleasure and walking at night was more enjoyable than during the daytime [37].

#### 3.4.2. Appropriateness of Walking Space

Vast open spaces for walking, like wide sidewalks and large open land covered with grass encouraged walking, while a lack of such spaces, such as narrow sidewalks, discouraged it [30,39,41,42]. Barriers on sidewalks, like parked cars, signs, furniture, trees, and shops occupying part of the sidewalk reduced the useable width of sidewalks and made the participants feel there was insufficient space for walking [30]. Small parks were found to discourage dog walking [41]. The good maintenance and quality of sidewalks were incentives for walking [30,42], while inadequate maintenance discouraged it [37,41,43]. A connected network of walking routes or recreational green spaces encouraged walking [39,41] and the lack of connections among walking spaces, including the absence of sidewalks, discouraged walking [41].

#### 3.4.3. Inadequate Public Transportation

The lack of integration of public transportation with walking routes, such as a lack of access to public transportation stations, discouraged walking to stations [39,42]. Lack of information about the times of public transport arrivals was also perceived as a barrier to the use of public transportation [42].

#### 3.4.4. Sounds in the Environment

Noise pollution caused by car heavy traffic or construction discouraged walking [8,30]. Traffic noise disturbed the contemplations of walkers, while an atmosphere of calm and quiet in the environment made it possible to think while walking [38]. Green elements reduced the discouraging influence of noise pollution [30].

A male participant cited listening to music as a method of reducing the effect of environmental noise and improving thinking conditions while walking [8]. A female participant cited awareness of environmental sounds as preferred over music, because lack of awareness of the sounds of an environment produced feelings of insecurity. The female participant preferred listening to music in uncrowded and relatively quiet spaces [38].

#### 3.4.5. Facilities

Freshening facilities such as cafes and beverage shops encouraged both recreational walking and walking for transportation because participants could rest and be refreshed during walking. The availability of parking spaces at the destination also impacted on the decision to walking [30,42]. 

#### 3.4.6. Natural Conditions

Bad weather and steep slopes were considered as barriers to transportation walking [30,40,43]. Walking with children in cold weather was difficult [40]. Green natural elements with clean air and shade were found to mitigate the negative influence of natural conditions like hot weather on walking [30,41].

## 4. Discussion

In qualitative studies, walking was considered to be a complex context bond and a socially and culturally sensitive phenomenon; consequently, in this meta-synthesis, social environment factors, as well as physical environment factors that influence walking, were explored. However, in quantitative systematic reviews [4,6,7,23], the social environment factors influencing walking were not considered.

Despite the quantitative systematic reviews [4,6], the sense of insecurity and of inadequate safety were explored separately and their physical and social subsets were defined based on the experiences of the participants in the qualitative studies. Some subset factors that impact on insecurity have been tested in studies about the relationship between the environment and crime [49,50]. However, these studies have not examined the relation between insecurity and walking. It is recommended that future quantitative studies examine the impact of obtained subset factors of safety and security on walking.

Despite the quantitative systematic reviews [4,6], all studies emphasized the influence of environmental aesthetics on diverse types of walking, including walking for transportation and recreational walking. One reason for the differences in study results could related to differences in the perception of aesthetics. The factors that constitute the concept of environmental aesthetics were derived from the qualitative studies; however, some, such as the presence or absence of details, and legibility and order were not considered in quantitative systematic reviews [4,6]. Examination of these factors in future quantitative studies is recommended.

In quantitative systematic reviews, the influence of distance on walking for transportation was emphasized, but this was not related to recreational walking [4,6]. The results of the meta-analysis showed how distance influenced various types of walking. In recreational walking, the pleasure of the environmental aesthetics, and being with others prompted walkers to choose longer paths and expend more time on walking. In walking for transportation, a limited time was considered suitable for walking and participants preferred to choose other modes of transportation if an extended length of time was needed.

Space syntax theory states that there will be higher movement density in the streets with higher connectivity [51,52]. The relationship of street connectivity with the amount of walking has been examined in quantitative systematic reviews [4,6] and there are discrepancies in the results. This meta-synthesis found that the connectivity of walking spaces was important to participants. Additionally, the necessity of making many stops and waiting at multiple intersections while walking as a consequence the walking spaces being broken up by highly-connected streets was a negative influence on the walking experience. Consequently, more studies examining the effect of connectivity of walking spaces and streets on walking are required.

A comparison of the results of this meta-synthesis with quantitative systematic reviews showed that qualitative studies could add detailed information on what and how environmental factors influence walking. Combining the results of this qualitative systematic review with those of quantitative studies can improve understanding of walking as a complex phenomenon and help to recommend strategies to improve the physical activity and health of communities and individuals.

Further discussions about results of this study are presented below. In relation to the sense of inadequate safety, the main concern was fear of accidents, which increased when walking with children in poor neighborhoods. Studies on the relationship between the sense of inadequate safety and walking with children in areas with middle class and upper class neighborhoods is recommended.

In relation to the built environmental aesthetics, uncleanliness was only mentioned in areas of low economic status. Observing an industrial environment was found to be pleasant by Davies et al. [42] and unpleasant by Bean et al. [39]. This contradiction can be explained in terms of the walking context and purpose. Davies et al. found that a historical-industrial environment in the countryside along a recreational walking route was perceived as pleasant for leisure walking. Bean et al. found that an industrial environment within a city was experienced as unpleasant during walking for transportation [39,42].

The positive influence of natural elements on walking, except dog walking, was consistently confirmed. Natural elements were also found to mitigate the negative influence of some factors on the walking experience. The presence of trees and natural light were emphasized for transportation walking and natural scenery was emphasized for recreational walking. The influence of natural elements on social relations was shown. The psychological benefits of natural elements were emphasized.

In relation to have a companion for walking; in Bostock’s study, mothers had experienced walking with children as a stressful activity [40], while Davies et al. found it to be a pleasant experience [42]. Participants in Bostock’s study included mothers from social minorities who could not afford to own a personal car and preferred walking over public transportation to save money [40]. They mostly walked for daily shopping from local stores. The walking context was also a disadvantaged area and the children were under school age. Generally speaking, these mothers had no choice except walking with their children. Davies et al. stated that participants had spoken of their recreational walking experiences and expectations in walking routes and that most of the participants were 45 to 60 years of age [42]. It appears that different reasons for walking, different environmental factors, and probably the age of children led to such contradictory results. Further studies on the influence of economic conditions on various types of walking with children or alone are recommended.

Middleton [37] reported an unfavorable social ambience in an environment lacking aesthetic qualities. Bean et al. demonstrated a relation between factors such as aesthetic quality as encouraging both interactions with others and walking [39]. It is possible that factors such as environmental aesthetics influence the positive or negative experiences of social ambience. Further studies are required to understand the relationship between factors, such as environmental aesthetics and seeing and interacting with others while walking.

Neighborhood scale studies [40,43] were different from other studies according to level of income. It appears that differences between findings of these studies and those of other studies resulted from the income level rather than scale variation. The use of focus groups for gathering data allows identification of more environmental factors influencing walking in comparison with the use of individual interviews.

The authors faced some limitations when synthesizing the studies’ results. No description was provided about some factors influencing the walking experience. Some factors were also compound with high level of abstraction, but their subcategory concepts had not been described. In such cases, it was difficult to analyze and interpret the factors and compare them with similar cases.

## 5. Conclusions

The purpose of this study was to identify the environmental factors influencing walking and explore how they influence adult walking experience in qualitative studies. Environmental factors influencing the walking experience were “safety and security”, “environmental aesthetics”, “social relations”, and “convenience and efficiency”. In this meta-synthesis, the variety of both social and physical factors influencing walking were explored. The social factors and some physical factors have not been considered in recent quantitative systematic reviews.

The present study examined how environmental factors impacted on walking. These environmental factors were found to relate to one another. Some factors enhanced or hindered the impact of other factors on walking. Convenience and efficiency enhanced the impact of social relations on walking in some aspects. Environmental aesthetics and social relations could hinder the influence of convenience and efficiency on walking experience. Conditions like income level could influence the impact of the environment on walking. The results of this meta-synthesis present descriptions of some discrepancies between the results of quantitative systematic reviews. In conclusion, this meta-synthesis added more variety and depth to the previous knowledge of the influence of the environment on walking.

The results of this study suggest some strategies to enhance the walking experience. These included proper maintenance and adequately wide walking places such as sidewalks, increasing the population density and providing mixed land use to shorten walking distances. Combining natural elements and green spaces in the walking environment, providing sufficient variety, details, and legibility in the design of buildings and public spaces, managing and protecting view corridors from walking spaces to picturesque places are additional suggestions. The proper lighting of walking places and providing sufficient crosswalks on streets should be a priority for pedestrians. As the population density increases, traffic and insufficient walking spaces should be considered.

It is recommended to carry out quantitative studies to examine the effect of the new environmental factors obtained from qualitative studies on walking. Further qualitative studies under different contexts using more sophisticated methods (such as grounded theory) are required to understand the interrelationship of environmental factors and their influence on the walking experience. In addition, conducting more qualitative research focused on issues like the influence of income level on the relation between the environment and walking experience and differences in the influence of the environment on walking alone in comparison with walking with others are recommended. The focus group method for gathering data is suggested to obtain more environmental factors influencing the walking experience.

## Figures and Tables

**Table 1:** Key words of systematic search.

Groups of Key Words	Key Words
Walking key words	walk, walking
Physical environment key words	environment, environmental, urban form, urban landscape, street scape, walkability, equipment, public policy, destination, sidewalk, pavement, footpath, trail, safety, city planning, urban planning, urban design, community design, neighborhood, enabler, motivator, facilitator, barrier, impediment, constraint, pedestrian, facility, infrastructure, space, land use, street connectivity, aesthetics, park, outdoor, sprawl, housing, recreation, traffic, residence characteristics, residential, convenience, behavioral context, situational factor
Qualitative key words	Interview, qualitative, findings, theme, grounded theory, grounded study, grounded research, grounded analysis, framework approach, focus group, ethnography, phenomenology, thematic, content analysis, narrative analysis, hermeneutic, action research, discourse analysis, observational method, fieldwork, ethnonursing, life story, women’s story, emic, etic, heuristic, semiotic, field study, field research, biographical method, conversation analysis, documentary analysis, lived experience, life experience, data saturation, participant observation, social construct, experiential, purposive sample, theoretical sample, theoretical saturation, constant comparative, key informant, humanistic, existential, paradigm, qualitative validity, personal experience, life world, category, constant comparison, postmodern, post structural, feminism, interpretation, co-operative inquiry, human science, open-ended account, unstructured account

**Table 2:** Features and summary of main findings of included studies.

Author and Date	Focus/Aim of the Study	Types of Walking	Data Collection	Data Analysis	Summary of Main Findings (Environmental Factors Influencing on Walking)
Bean et al., 2008 [39]	Understand the attitudes toward walking and driving	Non-specific types of walking	Focus group	Abductive	-Social pressure from other parents to walk more with children -Facilitators of recreational walking -Valuable places for walking for transport -Barriers to walking -Factors that encouraging both walking and social interaction
Bostock, 2001 [40]	Exploring the experience of walking in condition of carelessness in a low-income area	Non-specific types of walking	Individual interview	Abductive	-Obligation feeling of walking in an unclean and socially disadvantaged area -Vacant house and bad maintenance of amenities as discouraging factors -Barriers to walking with children
Burgoyne et al., 2007 [43]	Exploring the attitudes towards walking	Non-specific types of walking	Focus group	Inductive	-Security -Feelings of being neglected -Social factors -Physical environmental factors
Cutt et al., 2008 [41]	Understanding facilitators and barriers to dog walking	Dog walking	Focus group	Abductive	-Physical environmental barriers/facilitators -Social environmental barriers/facilitators
Darker et al., 2007 [8]	Understanding the meaning of walking experience	Non-specific types of walking	Individual interview	Deductive	-Long distance as a barrier to walking -Motivators of recreational walking -The influence of natural environment on pleasant walking -Limit of time as a barrier to walking except for enjoyable recreational walking
Davies et al., 2012 [42]	Identifying the attitudes and preferences of recreational walkers	Recreational	Focus group	Inductive	-Barriers to recreational walking -Preferences whilst walking -Dislikes whilst walking -Transport to and from recreational walks -Variety in designing a trail
Degeling & Rock, 2013 [44]	Exploring the experience of dog walking in public spaces	Dog walking	Individual interview	Abductive	-Dog as a facilitator to keep family together in walking -Sense of more security in walking with dog
Ferrer et al., 2015 [30]	Understanding the influence of built environment on walking and its experienced pleasantness	Transportation	Focus group	Abductive	-Safety from crime -Traffic safety -Walking facilities -Aesthetics -Convenience and other perceptions
Middleton, 2009 [37]	Exploring the walking experience and understanding the influence of time and space on walking	Non-specific types of walking	Individual interview and diaries	Deductive	-Choosing more direct routes under time limitation conditions -Busy street, poor quality of pavement and uncleanness, unfriendly social environment and waiting time of street crossings more important than saving time -More enjoyable walking at night in comparison with day time
Middleton, 2010 [38]	Exploring the walking experience and understanding the influence of built and social environment on walking	Non-specific types of walking	Individual interview and diaries	Deductive	-Preference for continuity of movement in transportation walking -Uncleanness and noises as barriers to thinking while walking -Importance of having auditory awareness

**Table 3:** Environmental factors which influenced the walking experience in three levels of themes, sub-themes and concepts (plus or minus signs refer to positive or negative influence on walking).

Themes	Sub-Themes	Concepts
Safety and Security	Sense of Insecurity	Absence of others and night time (−) Darkness (−) Closed shops, parking lots or vacant land (−) Presence of antisocial individuals, groups or behaviors (−) Walking with a dog (+)
	Sense of inadequate safety	Pedestrians crossing (wide streets and roundabouts, pedestrian traffic light without counter) (−) High speed or misbehavior of cars or bicycles (−) Un-partitioned sidewalks with cycle or car lanes (−) Width of streets (too wide; too narrow) (−) Some animals (−)
Environmental Aesthetics	Built Environment Aesthetics	Sights (picturesque sights (+); undesirable views (−); industrial environment (+/−)) Variety (+) Details (presence of details (+); lack of details (−)) legibility and order (sufficient legibility and order (+), extreme legibility and order (−)) Uncleanness (−)
	Natural Elements	Presence of trees (+) Presence of water (+/−) Natural light and fresh air (+) Spectacular landscape (+)
Social Relations	Being with Others	Presence of people around (crowded places (−); not crowded places (+)) Having a companion for walking (+/−) Seeing and interacting with others (+/−)
	Public perception	The culture of using cars (−) Social pressure for more walking with children (+) Sense of abandonment (in low income areas) (−)
Convenience and Efficiency	Time of walking	walking duration (long distance (−); short distance (+); waiting time on junctions (−)) Time limit (maximum time limit, shortage of time) (−) Time condition of walking (night) (+)
	Walking Spaces Appropriateness	Perceived amount of walking space (vast space (+); shortage of space (−)) Maintenance and quality of walking routes (proper maintenance (+); unsuitable maintenance (−)) walking spaces connectivity (connectivity of sidewalks and recreational green routes (+); lack of connection of sidewalks (−))
	Inefficient public transportation services	Lack of combination of public transportation with walking (long distances with stations) (−) Lack of information about arrival times (−)
	Sounds in the environment	Irritating noises (traffic, construction) (−); quiet (+)
	Facilities	Freshening facilities (+) Parking spaces for cars (−)
	Natural conditions	Slope (−) Weather (−)
